# Gender differences in outcomes among patients with heart failure and preserved ejection fraction: A retrospective cohort study

**DOI:** 10.6026/973206300214355

**Published:** 2025-12-15

**Authors:** Vidhi Jagdishkumar Sonara, Rekha Joe, Glenn Jo, Tania George, Vinupriya Talari, Anand Anilkumar

**Affiliations:** 1 Department of Respiratory Medicine, Medical Officer, Government Medical College, Surat, Gujarat, India; 2 Department of Interventional Radiology, GMC, Kalamassery, Kochi, Kerala, India; 3 Department of General Medicine, RMO, Kerala State Medical Councils, Kunnukuzhy, Thiruvananthapuram, Kerala, India; 4 Department of Cardiology, Baby Memorial Hospital, Kozhikode, Kerala, India; 5 Department of Cardiology, Yashoda Hospitals, Hyderabad, Telangana, India; 6 Department of General Medicine, Aster MIMS, Kannur, Kerala, India

**Keywords:** Heart failure, HFpEF outcomes, hospitalization, death, gender differences, preserved ejection fraction

## Abstract

Gender differences in the clinical presentation and outcomes of heart failure with preserved ejection fraction (HFpEF) remain inadequately characterized. This retrospective cohort study evaluated 146 HFpEF patients to compare baseline features and one-year outcomes between males and females. Female patients were more frequently obese or hypertensive, whereas male patients more often had coronary artery disease. Despite these differing risk profiles, females exhibited lower mortality and all-cause hospitalization rates at one year. These findings underscore meaningful gender-based variations in HFpEF and support the need for gender-sensitive strategies in clinical management and prognostication.

## Background:

Heart failure with preserved ejection fraction (HFpEF) accounts for over half of all heart failure diagnoses and is increasingly recognized as an important issue in cardiovascular medicine [1]. HFpEF is primarily characterized by diastolic dysfunction, where the left ventricle has impaired relaxation and stiffness, increasing filling pressures even when systolic function remains preserved. HFpEF primarily affects older adults, who often have multiple co-morbidities (e.g. hypertension, obesity, diabetes, chronic kidney disease), which increases the complexity of its pathophysiology [2]. Recent literature identifies important gender differences in HFpEF - those related to etiology, clinical presentation and disease progression. For example, women with HFpEF are more likely to present with obesity, hypertension and preserved systolic function; men with HFpEF however, often experience more left ventricular remodeling, ischemic heart disease and more myocardial tissue fibrosis [3]. Differences can also be seen in symptomology, whereby women report as having more exercise intolerance and more dyspnea and men report as experiencing more overt symptoms of volume overload. Hormonal influences, body composition and vascular stiffness are suggested mechanisms describing these sex-specific differences, while precise mechanisms remain to be determined [4]. Even though distinctions have been recognized, little is known about the role of gender in long-term clinical outcomes in HFpEF. Evidence of rates of hospitalization, deaths, functional decline and quality of life by sex are mixed [5]. Some studies report that while women may survive better, she may experience a higher burden of symptoms; while men may have a worse prognosis due to ischemic heart disease and adverse remodeling [6]. Understanding these differences are important in developing plans of care and targeted therapy because traditional heart failure therapies may not be effective within HFpEF populations [7]. Real-world clinical data may be especially valuable for our understanding of these topics, since clinical trial populations often underrepresent older patients and women-compositional groups often contributing the most to the HFpEF population [8]. Observational studies using retrospective registries and electronic health records (EHRs) offer additional information on the potential for genderbased differences in HFpEF and outcomes over a year, including hospitalization and death rates [9]. Understanding gender differences may help clinicians create a more customized plan of care that optimizes resources and promotes patient-centered care, all within the diverse HfpEF population [10]. Therefore, it is of interest to describe gender differences in clinical characteristics and one-year outcomes among patients with heart failure with preserved ejection fraction in a real-world cohort.

## Materials and Methods:

Adult patients (aged 18 years or older) with symptomatic heart failure and an echocardiogram-documented left ventricular ejection fraction (LVEF) of at least 50%, were included in this retrospective cohort study. Over a 24-month period in a tertiary care hospital setting, patients were identified by computerized medical record data. A total of 146 patients met criteria for inclusion and had complete baseline characteristics and one-year follow-up data available. Patients were excluded if they had valvular heart disease, heart failure with reduced ejection fraction (HFrEF), or incomplete records of baseline characteristics. We collected the following data: demographics, medical comorbidities of hypertension, diabetes mellitus, coronary artery disease, atrial fibrillation and obesity, medications, echocardiographic parameters and laboratory values at baseline. The primary outcomes were all-cause hospitalization and mortality within one year of diagnosis. Secondary outcomes included NYHA functional class, NT-proBNP levels and medication usage patterns. Data were analyzed using SPSS version 26. Continuous variables are reported in terms of mean ± standard deviation and were compared using Student's t-test. Categorical variables were compared using Chi-square or Fisher's exact test as appropriate. Cox regression was performed to identify independent predictors of mortality with gender as an important covariate. A p-value < 0.05 was considered statistically significant.

## Results:

Among 146 patients diagnosed with HFpEF, 88 (60.3%) were female and 58 (39.7%) were male. Female patients were older and had a higher prevalence of hypertension and obesity, whereas males had more coronary artery disease and smoking history. No significant difference was observed in left ventricular ejection fraction (LVEF) or NT-proBNP levels between genders. Female patients presented with higher NYHA functional class, though medication usage at discharge was comparable across genders. One-year all-cause hospitalization was higher in males (45.5%) than females (31.8%) and mortality was significantly greater in males (20.7% vs 9.1%). Multivariate Cox regression analysis identified male gender, coronary artery disease and NYHA Class III-IV as independent predictors of one-year mortality. These findings suggest gender-based differences in clinical presentation and outcomes among HFpEF patients.

Table 1 (see PDF) shows that female patients were significantly older than males in this cohort. Table 2 (see PDF) shows that hypertension and obesity were more prevalent among females, whereas males had higher rates of coronary artery disease and smoking history. Table 3 (see PDF) shows that average LVEF did not differ significantly between male and female patients. Table 4 (see PDF) shows that females presented with higher NYHA functional class at baseline compared to males. Table 5 (see PDF) shows that NT-proBNP levels were slightly higher in females, but the difference was not statistically significant. Table 6 (see PDF) shows that usage of beta-blockers, ACE inhibitors/ARBs, diuretics and MRAs at discharge was comparable between genders. Table 7 (see PDF) shows that one-year all-cause hospitalization was significantly higher in males than females. Table 8 (see PDF) shows that one-year mortality was more than double in males compared to females. Table 9 (see PDF) shows that the primary causes of hospitalization in both genders were HF exacerbation or volume overload, followed by arrhythmias and acute coronary syndrome. Table 10 (see PDF) shows that in multivariate Cox regression analysis, male gender, coronary artery disease and NYHA Class III-IV were independent predictors of one-year mortality, while age was not.

## Discussion:

This retrospective cohort study reveals significant gender-based disparities in outcomes among patients with HFpEF. Although females constituted the majority of the cohort and were significantly older, males experienced worse one-year outcomes, including higher hospitalization and mortality rates [11]. These findings align with growing literature suggesting that HFpEF is not a uniform entity and that sex-specific differences play a critical role in disease progression and prognosis. Female patients were more likely to present with hypertension, obesity and advanced NYHA functional class, reflecting the typical HFpEF phenotype observed in women older, hypertensive and obese [12]. However, despite this more symptomatic presentation, females had better outcomes than their male counterparts. Male patients, conversely, had higher rates of coronary artery disease and smoking history, both of which likely contributed to their poorer prognosis [13]. These results support prior studies showing ischemic heart disease as a key modifier of adverse events in HFpEF, particularly among men. The higher mortality and hospitalization rates in males may also reflect more significant myocardial remodeling and subclinical systolic dysfunction not fully captured by preserved LVEF alone [14]. The multivariate analysis confirmed that male gender, CAD and higher NYHA class were independent predictors of mortality. This highlights the importance of aggressive risk stratification and CAD management, particularly in male HFpEF patients [15]. Despite similar medication usage across both sexes, outcome disparities persisted, suggesting that standardized treatment may not be equally effective across genders. The lack of gender-tailored therapeutic strategies may partly explain these differences. This study reinforces the urgent need to recognize and incorporate sex-specific pathophysiology into HFpEF management and clinical trials.

## Conclusion:

In this retrospective HFpEF cohort, females-despite being older and more symptomatic-demonstrated superior one-year survival and lower hospitalization rates compared with males. Male gender, coronary artery disease, and advanced NYHA class emerged as independent predictors of mortality. These findings underscore the need for gender-specific risk stratification and individualized management strategies to optimize outcomes in HFpEF.
